# Supplementary material for: REGULATORY T CELLS PROTECT AGAINST ABERRANT REMODELING IN A MOUSE MODEL OF PULMONARY FIBROSIS
Source: bioRxiv. 2025 Jul 5:2025.07.02.662777. Preprint. [Version 1] doi: 10.1101/2025.07.02.662777 (PMC12236608; doi:10.1101/2025.07.02.662777)
Supplement: Supplement 1 [file media-1.pdf]

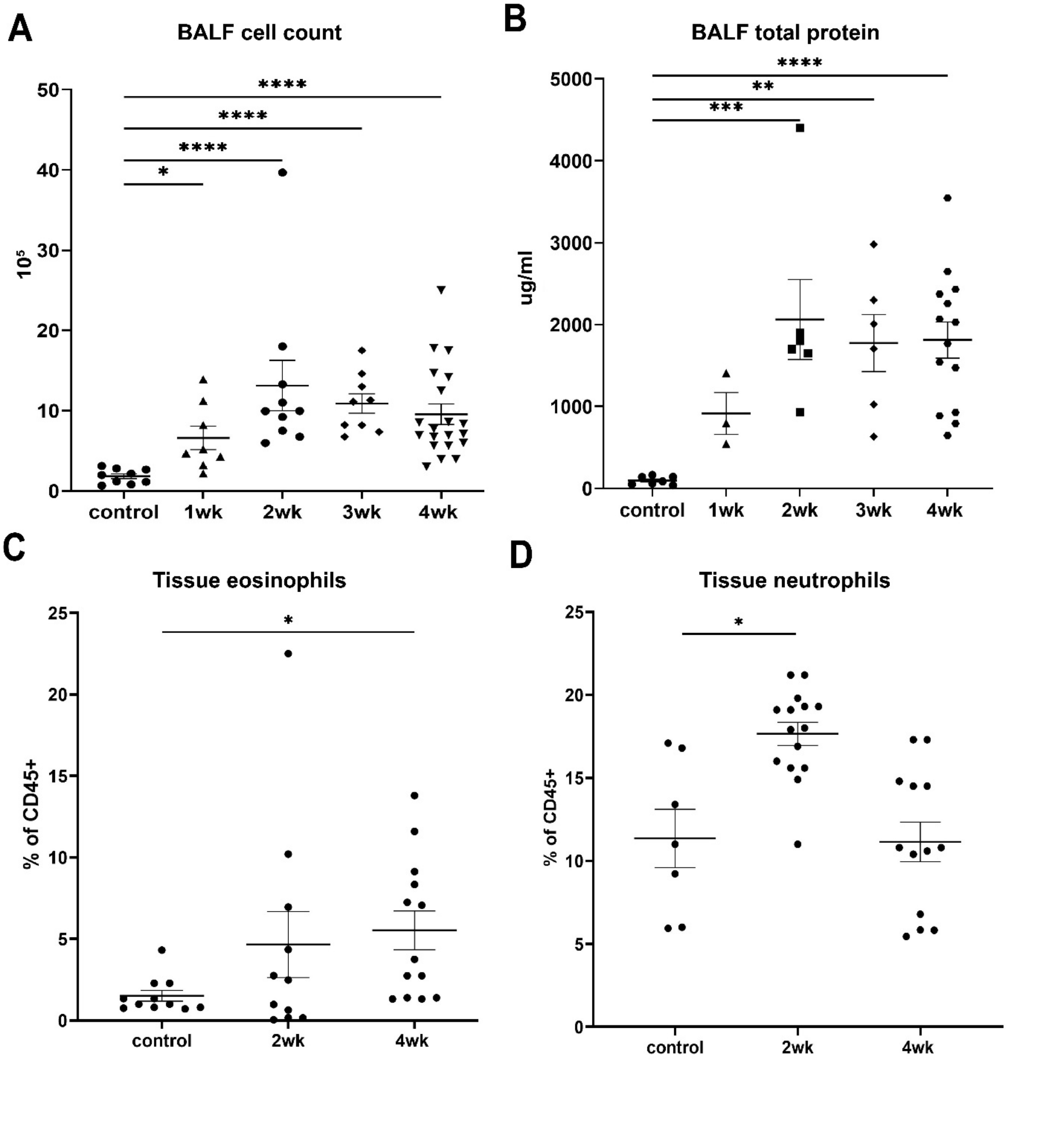

Supplemental figure 1: AT2-specific lung injury induces alveolitis Bronchoalveolar lavage fluid (BALF) collected at defined time points after two oral doses of tamoxifen in SP-C^{I73T} and control mice showed increased (A) total cell counts and (B) protein concentrations. Flow cytometric analysis of lung tissue at 2- and 4-weeks post-injury revealed elevated myeloid cell populations, including (C) eosinophils and (D) neutrophils. Statistical analysis was performed using one-way ANOVA (*$p < 0.05$, **$p < 0.005$, ***$p = 0.0009$, ****$p < 0.0001$). SP-C^{I73T}, n = 3–20; control, n = 7–11.

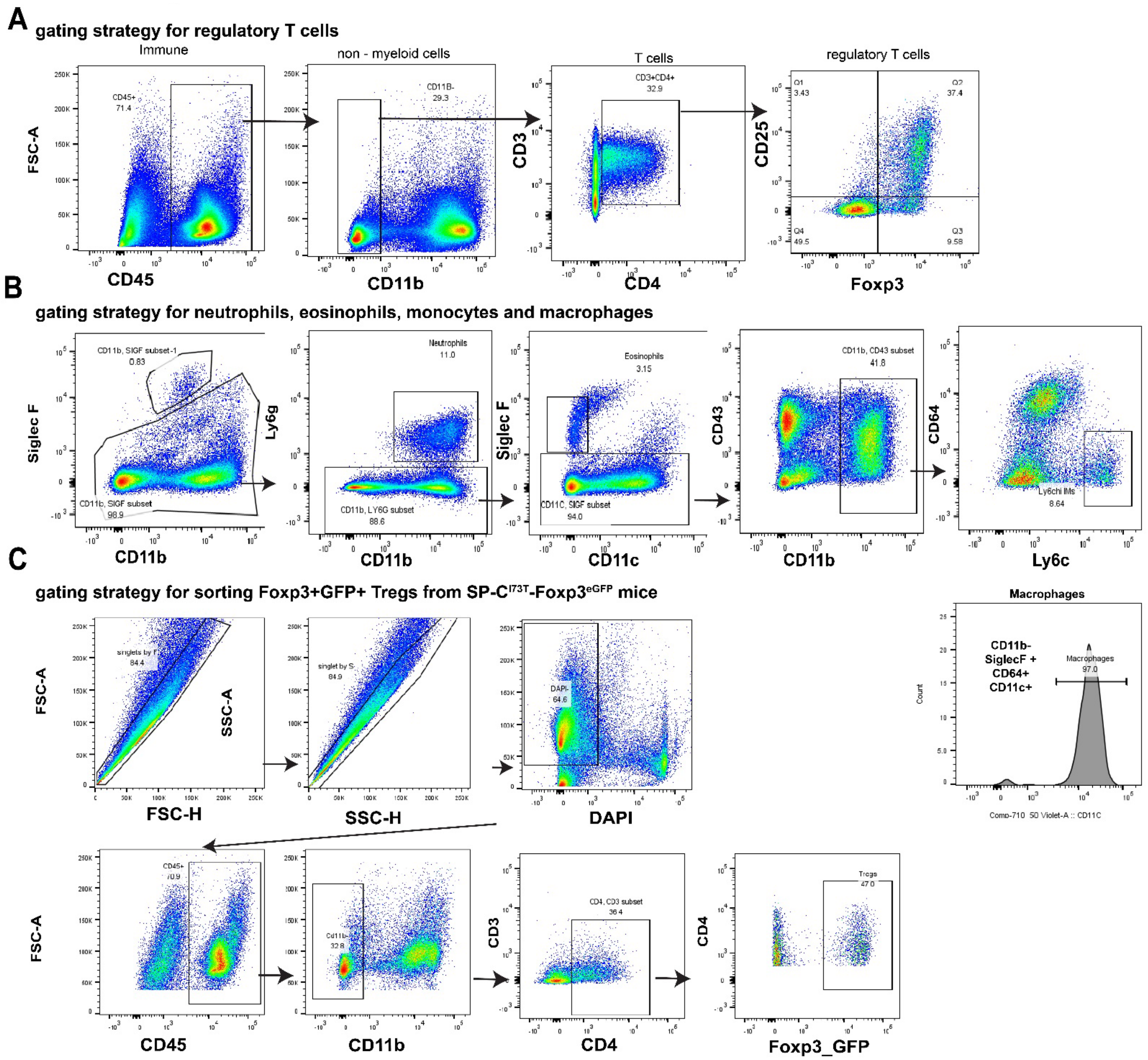
A
gating strategy for regulatory T cells
Immune
non - myeloid cells
T cells
regulatory T cells
CD45+
71.4
CD11B-
29.3
CD3+CD4+
32.9
Q1
3.43
Q2
37.4
Q4
49.5
Q3
9.58
FSC-A
CD45
CD11b
CD3
CD4
CD25
Foxp3
B
gating strategy for neutrophils, eosinophils, monocytes and macrophages
CD11b, SIGF subset-1
0.83
CD11b, SIGF subset
98.9
Neutrophils
11.0
CD11b, LY6G subset
88.6
Eosinophils
3.15
CD11C, SIGF subset
94.0
CD11b, CD43 subset
41.8
Ly6chi IMs
8.64
Siglec F
Ly6g
CD43
CD64
CD11b
CD11c
Ly6c
C
gating strategy for sorting Foxp3+GFP+ Tregs from SP-C^{I73T}-Foxp3^{eGFP} mice
singlets by F
84.4
singlet by S
84.9
DAPI-
64.6
FSC-A
SSC-A
FSC-H
SSC-H
DAPI
Macrophages
CD11b-
SiglecF +
CD64+
CD11c+
Macrophages
97.0
Count
Comp-710_50 Violet-A :: CD11C
CD45+
70.9
Cd11b-
32.8
CD4, CD3 subset
36.4
Tregs
47.0
CD45
CD11b
CD3
CD4
Foxp3_GFP

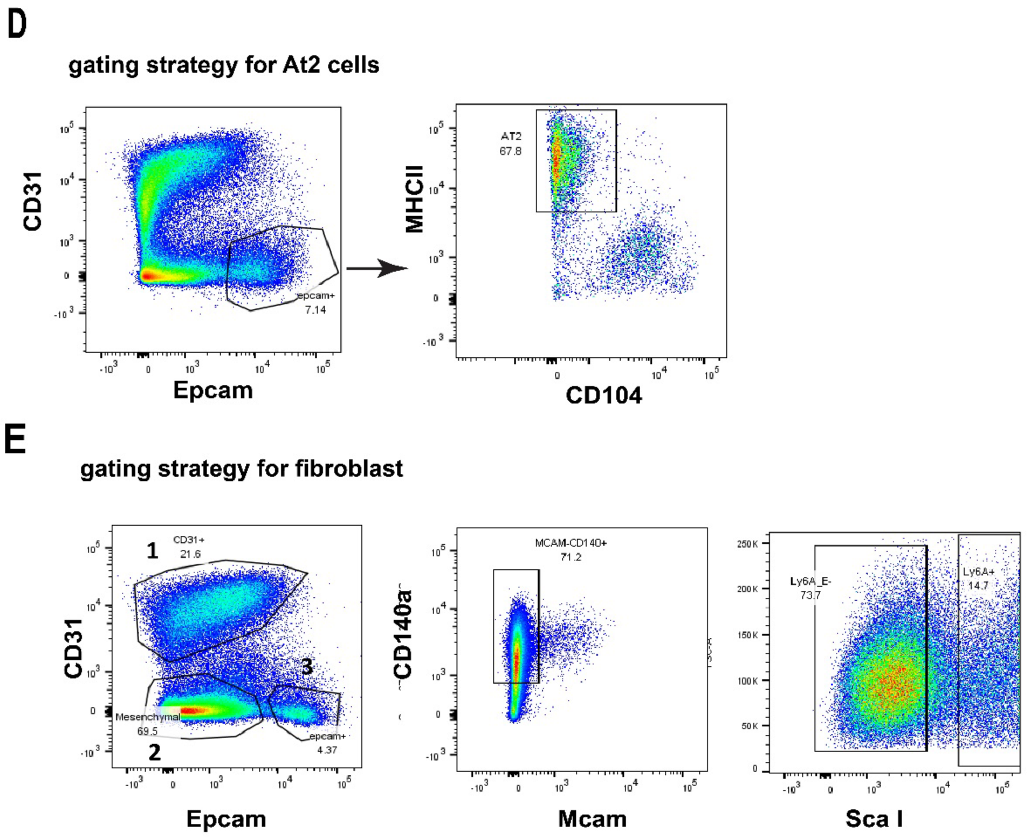

Supplemental figure 2: Gating strategy for lung immune, epithelial, and mesenchymal cell populations. Flow cytometric gating and phenotyping were used to identify (A) regulatory T cells, (B) neutrophils, eosinophils, monocytes, and macrophages in SP-C^{I73T} mouse lungs, and (C) regulatory T cells in SP-C^{I73T}Foxp3^{eGFP} mice. Epithelial and mesenchymal populations included (D) alveolar type 2 (AT2) cells and (E) adventitial fibroblasts.

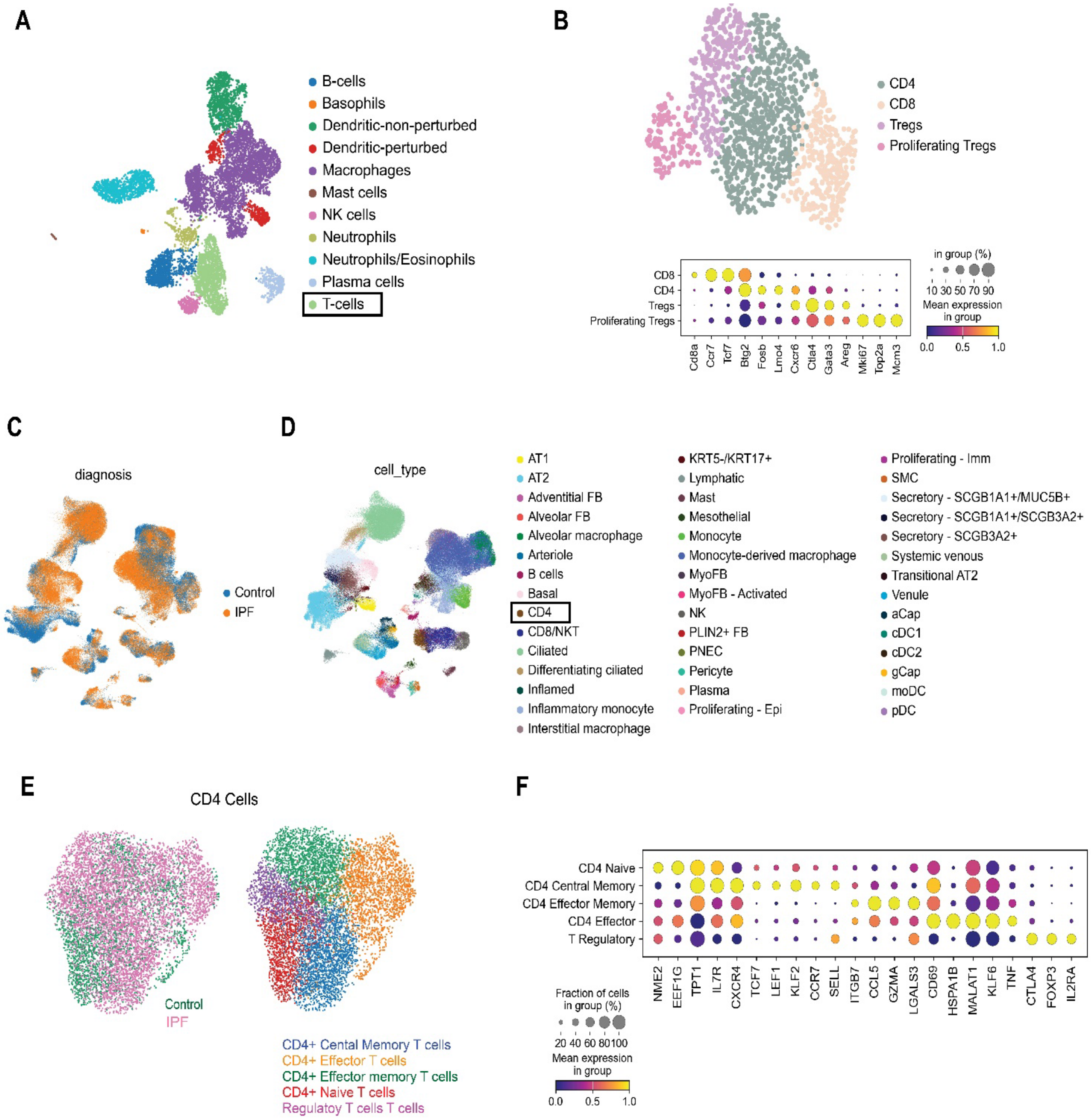

Supplemental Figure 3: Marker Genes Used to Identify Tregs in Murine and Human scRNA Data Sets A) Annotated UMAP of CD45 positive cells in previously published GSE234604 identifying major immune populations in *Sftpc*^{I73T} mouse lungs. B) Re-clustered UMAP of T-cells and gradient dot plot with marker genes used to define each T-cell subcluster. C) UMAP of IPF and control lungs from previously published human lung data set GSE227136 annotated by disease status D) Cell type annotation of GSE227136 as published by authors and highlighting a subset of cells that are mapped as CD4 T-cells. E) UMAP of CD4 T-cells in GSE227136 denoting disease status and newly annotated subclusters of CD4 T-cells. F) Gradient dot plot of marker genes used to identify and annotate the subclusters of CD4 T-cells.

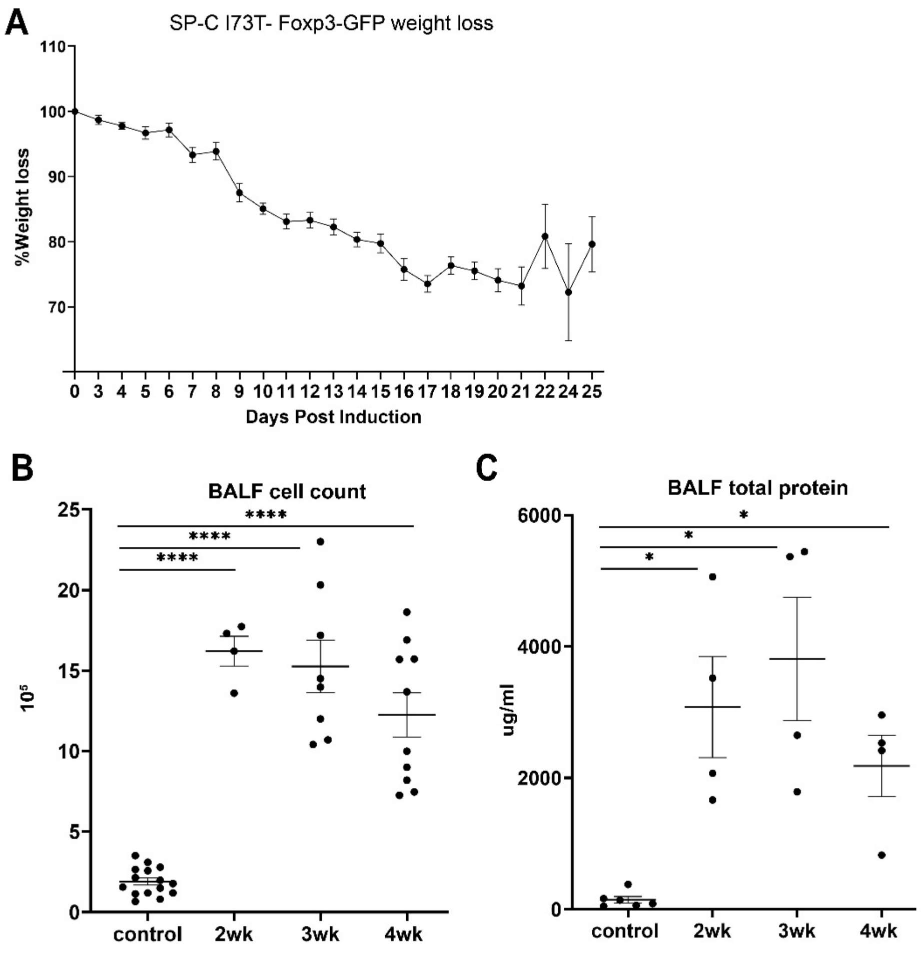

Supplemental figure 4: Generation of the SP-C^{I73T}Foxp3^{eGFP} mouse model to recapitulate AT2-specific lung injury. Tamoxifen administration in SP-C^{I73T}Foxp3^{eGFP} mice induced (A) progressive weight loss following AT2-specific mutant SP-C expression. Increased (B) total BAL cell counts and (C) protein concentrations at indicated time points indicate alveolitis and fibrosis, consistent with the phenotype observed in SP-C^{I73T} mice. Statistical analysis was performed using one-way ANOVA (*$p < 0.05$, ****$p < 0.0001$). SP-C^{I73T}, n = 4–10; control, n = 6–14**.**

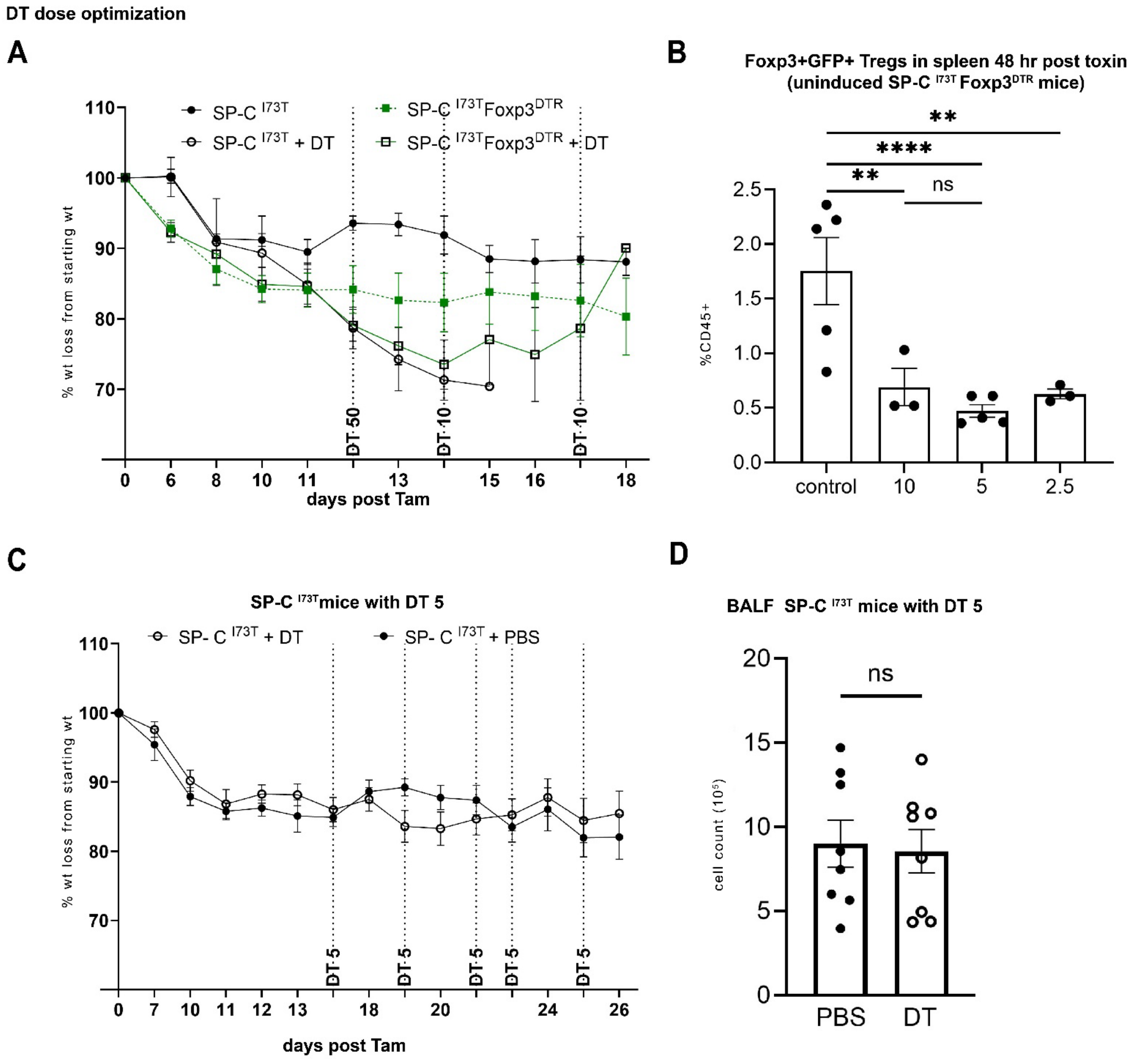

Supplemental figure 5: Diphtheria toxin dose (DT) optimization and toxicity studies in SP-C^{I73T} and SP-C^{I73T}-Foxp3^{DTR} mice (A) SP-C^{I73T}-Foxp3^{DTR} and SP-C^{I73T} mice received intraperitoneal DT injections (50, 10, and 10 µg/kg) beginning 12 days post-tamoxifen induction, with smaller doses administered every other day. Daily weight monitoring showed >30% weight loss in both groups. (B) Flow cytometric analysis of splenic CD45+ immune cells 48 hours post-DT administration (10, 5, or 2.5 µg/kg) assessed Treg depletion efficiency in SP-C^{I73T}-Foxp3^{DTR} mice. (C) Weight loss and (D) BALF total cell counts were measured in SP-C^{I73T} mice treated with either 5 µg/kg DT every other day or PBS. Statistical analysis was performed using one-way ANOVA (**p =0.001, ****p < 0.0001). SP-C^{I73T}Foxp3^{DTR} + DT n=7; SP-C^{I73T} + DT n=6; SP-C^{I73T}Foxp3^{DTR} + PBS n=4; SP-C^{I73T}+ PBS n =4.

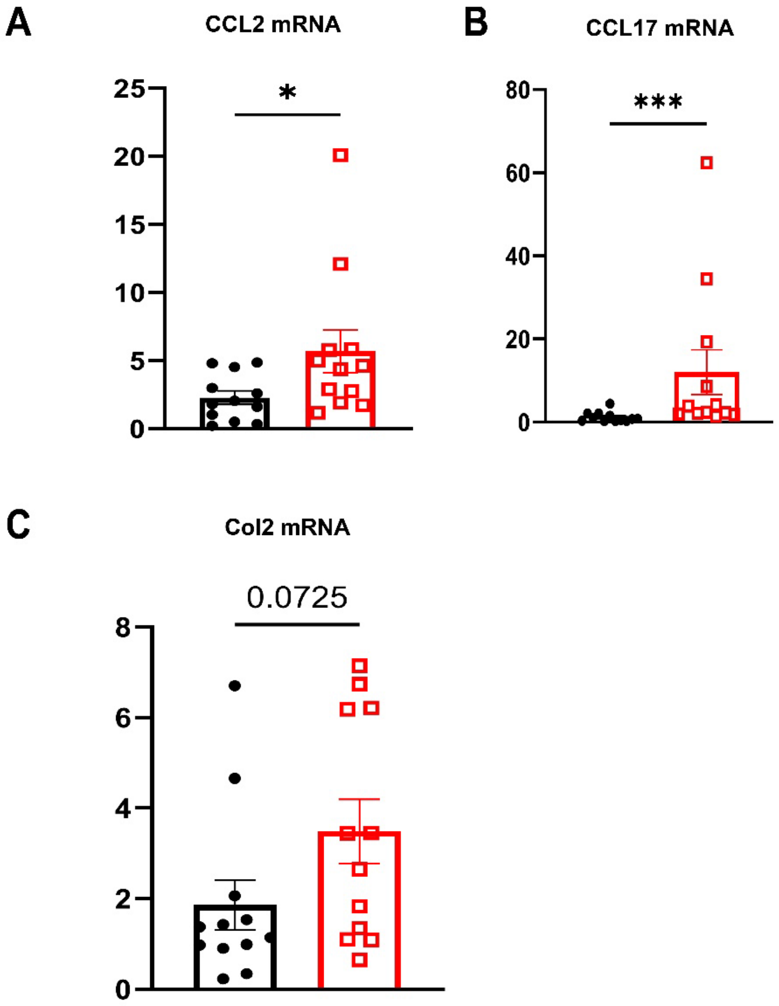

Supplemental figure 6: Treg depletion during the fibrogenic phase exacerbates disease in SP-C^{I73T}Foxp3^{DTR} mice. Mice received intraperitoneal injections of diphtheria toxin (5 µg) or PBS every 48 hours, starting on day 17 post-tamoxifen induction. On day 26, whole lung tissue was collected from DT-treated (n = 9) and PBS-treated (n = 9) SP-C^{I73T}Foxp3^{DTR} mice for gene expression analysis by qRT-PCR, pro-inflammatory chemokines (A) *Ccl2* and (B) *Ccl17*, which mediate monocyte and macrophage recruitment, as well as (C) the collagen-associated gene *Col2a1*, were elevated in DT-treated mice. Statistical analysis was performed using Unpaired t-test *p<0.05 *** p<0.001.

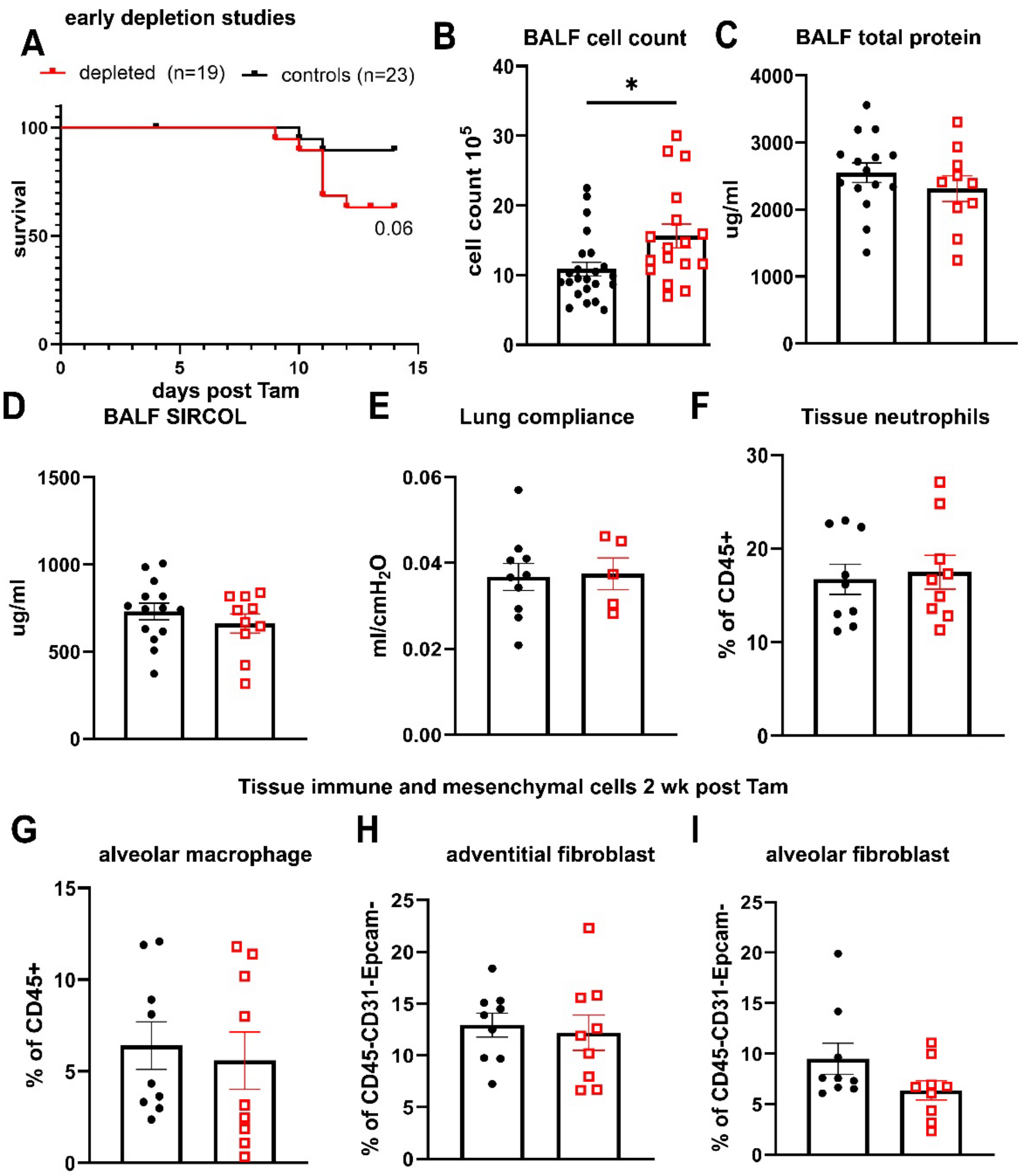

Supplemental Figure 7: Early Treg depletion does not alter fibrotic endpoints in SP-C^{I73T}Foxp3^{DTR} mice following tamoxifen induction. Mice received intraperitoneal injections of diphtheria toxin (DT; 5 µg) or PBS every 48 hours, starting 2 days prior to tamoxifen (Tam) induction (A) Kaplan–Meier survival curves of SP-C^{I73T}Foxp3^{DTR} mice treated with PBS (n=23) or DT (n=19); log-rank (Mantel–Cox) test, p = 0.0648. (B–C) Total bronchoalveolar lavage fluid (BALF) cell counts and protein levels at day 15 post-Tam induction (PBS: n=23; DT: n=19); unpaired t-test with Welch's correction, p = 0.01. (D-E) fibrotic endpoints at day 15: (D) BALF soluble collagen measured by Sircol assay (PBS: n=14; DT: n=10); (E) static lung compliance (Cst) (PBS: n=10; DT: n=5). (F–I) Flow cytometric analysis of lung immune (neutrophils, alveolar macrophages) and mesenchymal (adventitial and alveolar fibroblasts) populations on day 15 post-Tam (PBS: n=9; DT: n=9).

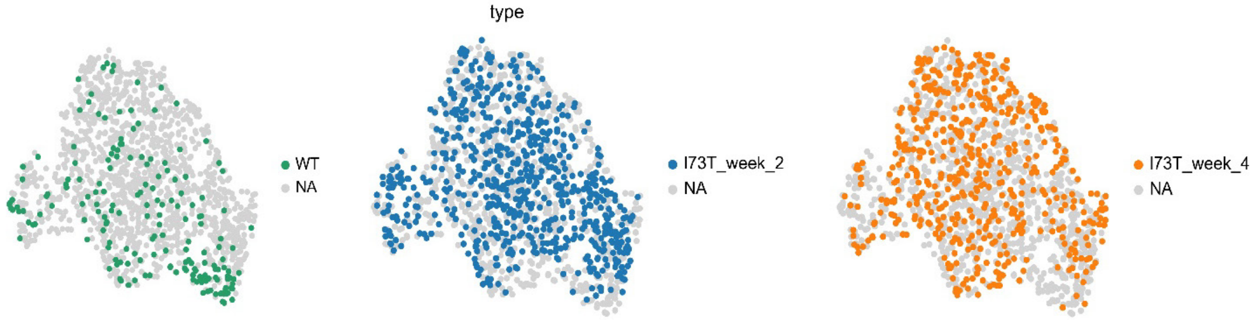

Supplemental Figure 8: Increased expression of T cell–specific genes in SP-C^{I73T} mice. Single-cell RNA sequencing (scRNA-seq) was performed on lungs from control and SP-C^{I73T} mice at 2- and 4-weeks post-tamoxifen induction (GSE234604), as described in Methods. (A) UMAP analysis of 1,341 CD3+ T cells reveals increased expression of T cell–specific genes following mutant SP-C expression. *n* = 2 mice per genotype per time point**.**

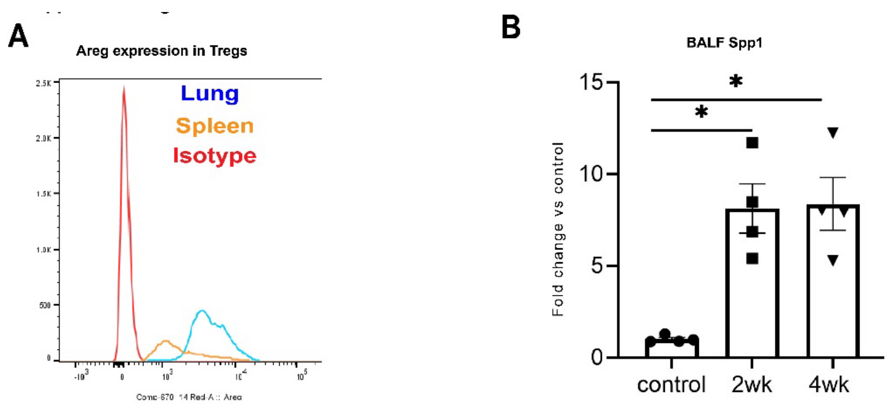

Supplemental Figure 9: Lung Tregs produce pro-repair growth factors in SP-C^{I73T} mice. (A) Foxp3-GFP+ Tregs were FACS-purified from lungs and spleens of SP-C^{I73T}Foxp3^{eGFP} mice 4 weeks post-tamoxifen induction, stimulated overnight with a cell stimulation cocktail (see Methods), and stained for intracellular Amphiregulin (Areg). Histograms show increased Areg expression only in lung-derived GFP+ Tregs following mutant SP-C expression. (B) BALF samples from control and SP-C^{I73T} mice collected at 2- and 4-weeks post-induction were analyzed for Osteopontin (Spp1), a protein involved in growth, repair, and IPF pathogenesis.

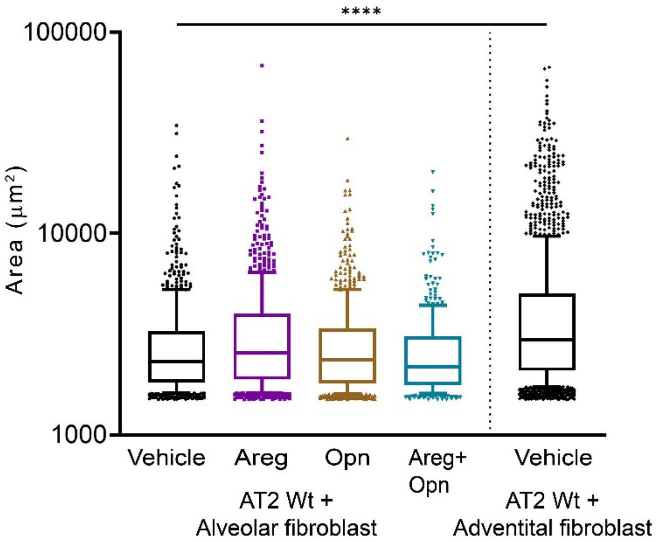

Supplemental Figure 10: Lung SP-C^{I73T} T regs selectively enhance adventitial, but not alveolar, fibroblast-supported alveolar organoid growth in vitro post injury Bi-cellular organoids containing FACS purified TdTom+ AT2 and ScaI+ adventitial fibroblast were cultured with either 100 nM Areg, 200 nM Opn, or Areg + Opn for 2 weeks. For comparison, tricellular organoids containing lung Tregs were prepared and co-cultured for 2 wks. Live cultures were imaged using EVOS FL Auto and images were quantified using ImageJ an "analyze particle" macro with minimum area threshold set to1500 μm^2. The experiment was independently repeated at least three times, each with three biological and three technical replicates ****$p<0.00005$ done by Ordinary one-way ANOVA.

Supplemental Table 1: Antibodies for Flow Cytometry				
Antibody	Fluor	Clone	Catalog Number	Manufacturer
CD45	BB515	30F-11	564590	Biolegend
CD45	APC	30F-11	103112	Biolegend
CD25	APC	PC61	561048	BD Biosciences
CD25	BV605	PC61	563061	BD Biosciences
Foxp3	PE	FJK-16s	12-5773-82	Thermofisher
Foxp3	APC	FJK-16s	12-5773-82	Thermofisher
CD3e	APC/fire 750	145-2C11	100361	Biolegend
CD3e	PE/Cy7	145-2C11	561100	BD Biosciences
CD4	BV786	GK1.5	5633331	BD Biosciences
GATA3	PE	TWAJ	12-9966-42	Invitrogen
Tbet	BV421	4B10	644815	BD Biosciences
Rorgt	BV21	Q31-378	562894	BD Biosciences
CD62L	PE/Cy7	MEL-14	104417	BD Biosciences
Tigit	PE/Cy7	1G9	142107	Biolegend
PD1	BV711	J43	744547	Biolegend
GITR	PE/Cy7	YGITR 765	120222	Biolegend
CD103	Percp cy5.5	2 E 7	121415	Biolegend
CD44	PE	IM7	553133	BD Biosciences
Epcam	BV711	G8.8	118233	Biolegend
Epcam	BV785	G8.8	118245	BD Biosciences
CD31	APC/Cy7	MEC13.3	102533	Biolegend
CD104	PE/Cy7	346-11A	123615	Biolegend
CD51	PE	RMV-7	104105	Biolegend
SiglecF	PEGF594	E50-2440	562757	BD Biosciences
CD11b	BV421	M1/70	101205	eBiosciences
CD11c	BV711	HL3	561022	Biolegend
Ly6g	AF700	1A8	561236	Biolegend
CD64	PE/Cy7	X54-5/7.1	139305	Biolegend
CD43	PE	S11	143205	Biolegend
Ly6C	BV510	HK1.4	128033	Biolegend
CD3e	BUV395	145-2C11	563565	BD Biosciences
Amphiregulin		Poly goat IgG	BAF989	R&D Systems
Biotin anti mouse Epcam		G8.8	118204	Biolegend
Biotin anti mouse CD140a		APA5	135910	Biolegend
Biotin anti mouse CD45		30F-11	103104	Biolegend
Biotin anti mouse CD31		MEC13.3	102504	Biolegend
Anti mouse CD3		17A2	16-0032-85	Thermofisher
Anti mouse CD28		37.51	16-0281-85	Thermofisher
live/dead	BUV395	viability dye	423107	Biolegend
live/dead	ef780	viability dye	65-0865-14	Thermofisher

Supplemental Table 2: Primer Sequences		
**Target**	**Forward**	**Reverse**
18S	CGGCTACCACATCCAAGGAA	GCTGGAATTACCGCGGCT
b-actin	GGCACCACACCTTCTACAATG	GGGGTGTTGAAGGTCTCAAC
Col3a	CTAAAGGGGAGATGGGCCTG	ATTATCGGGTTGGAGCGCAG
Col1a	GCAAGAGGCGAGAGAGGTTT	CAGTTCACACTCGTAGCCGT
Fgf7	CATGCTTCCACCTCGTCTGT	CAGTTCACACTCGTAGCCGT
mKi67	ACCATCATTGGACCGCTCCTT	GCCCTGATGAGTCTTGGCTA
Top2a	TACAGTGCTCAACCTCTGACG	GGGATCTCGTGTTGGGAAGG
Areg	GGTCTTAGGCTCAGGCCATTA	CGCTTATGGTGGAAACCTCTC
Ccl2	Mm00441242_m1	
Ccl17	Mm01244826_g1	

Supplemental Table 3: Antibodies for IF			
**Antibody**	**Catalog Number**	**Manufacturer**	**Dilution**
Brilliant Violet 605 Streptavidin	405229	Biolegend	1 in 200
APC Streptavidin	405207	Biolegend	1 in 200
Dapi (4',6-Diamidino-2-Phenylindole, Dihydrochloride)	D1306	Thermofisher	1 in 1000
CD39L1/ETPD2	AF5797	R&D Systems	1 in 200
RAGE	175410	R&D Systems	1 in 200
Foxp3	ab215206	abcam	1 in 200
Foxp3	14-5773-82	invitrogen	1 in 200
Npro2		In-house (refer to M&M)	1 in 200
Hoechst 33342	H3570	Invitrogen	1 in 1000
Goat anti Mouse - HRP	170-6516	BioRad	1 in 10000
Goat anti Rabbit - HRP	170-6515	BioRad	1 in 10000
AlexaFluor 647 Goat anti Rabbit	A32733TR	Invitrogen	1 in 400
AlexaFluor 488 Goat anti Rabbit	A32731	Invitrogen	1 in 400
AlexaFluor 488 Goat anti Rat	A11006	Invitrogen	1 in 400
AlexaFluor 674 Goat anti Rat	A21247	Invitrogen	1 in 400
